# The Endophytic Fungus Piriformospora Indica-Assisted Alleviation of Cadmium in Tobacco

**DOI:** 10.3390/jof7080675

**Published:** 2021-08-20

**Authors:** Zhenzhu Su, Yulan Zeng, Xiaoli Li, Anand Babu Perumal, Jianan Zhu, Xuanjun Lu, Mengdi Dai, Xiaohong Liu, Fucheng Lin

**Affiliations:** 1 Institute of Biotechnology, Zhejiang University, Hangzhou 310058, China; zzsu@zju.edu.cn (Z.S.); 12016090@zju.edu.cn (Y.Z.); 18846921667@163.com (J.Z.); luxuanjun595@163.com (X.L.); xhliu@zju.edu.cn (X.L.); 2 College of Biosystems Engineering and Food Science, Zhejiang University, Hangzhou 310058, China; xiaolili@zju.edu.cn (X.L.); anandbabu@zju.edu.cn (A.B.P.); 3 State Key Laboratory for Managing Biotic and Chemical Treats to the Quality and Safety of Agro-Products, Institute of Plant Protection and Microbiology, Zhejiang Academy of Agricultural Sciences, Hangzhou 310021, China; 11616058@zju.edu.cn

**Keywords:** endophytic fungi, *Piriformospora indica*, cadmium, tolerance, proteomics, glutathione

## Abstract

Increasing evidence suggests that the endophytic fungus *Piriformospora indica* helps plants overcome various abiotic stresses, especially heavy metals. However, the mechanism of heavy metal tolerance has not yet been elucidated. Here, the role of *P. indica* in alleviating cadmium (Cd) toxicities in tobacco was investigated. It was found that *P. indica* improved Cd tolerance to tobacco, increasing Cd accumulation in roots but decreasing Cd accumulation in leaves. The colonization of *P. indica* altered the subcellular repartition of Cd, increasing the Cd proportion in cell walls while reducing the Cd proportion in membrane/organelle and soluble fractions. During Cd stress, *P. indica* significantly enhanced the peroxidase (POD) activity and glutathione (GSH) content in tobacco. The spatial distribution of GSH was further visualized by Raman spectroscopy, showing that GSH was distributed in the cortex of *P. indica*-inoculated roots while in the epidermis of the control roots. A LC-MS/MS-based label-free quantitative technique evaluated the differential proteomics of *P. indica* treatment vs. control plants under Cd stress. The expressions of peroxidase, glutathione synthase, and photosynthesis-related proteins were significantly upregulated. This study provided extensive evidence for how *P. indica* enhances Cd tolerance in tobacco at physiological, cytological, and protein levels.

## 1. Introduction

Over the past few decades, increased anthropogenic activities, rapid industrialization, and modern agricultural practices have aggravated heavy metal pollution in the environment [1]. Large farmland areas have been contaminated with heavy metals due to the extensive use of pesticides, fertilizers, and contaminated water irrigation, posing a severe threat to crop safety and human health [2,3]. Cadmium (Cd) is one of the most toxic heavy metals in agricultural fields and raised various harmful effects on crop plants such as a decrease in yield, slow germination, leaf chlorosis, necrosis, etc. [4,5,6]. 

The application of heavy metal hyperaccumulators for phytoremediation is a sustainable solution. Various Cd hyperaccumulator plant species have been extensively investigated [7,8]. However, the hyperaccumulators have many disadvantages, such as low growth rate, small biomass, low commercial availability [9]. Therefore, some crop species with high commercial value, fast growth rate, high yield, and medium-heavy metal adsorption capacity provide new opportunities for hyperaccumulators. Tobacco (*Nicotiana tabacum* L.) is a well-known economic crop around the world. It has been applied as Cd phytoremediators for decades [10,11,12,13,14]. However, the Cd tolerance of tobacco is always limited to Cd concentration, Nicotiana varieties, exposure time, etc. [15]. Therefore, it is necessary to develop an innovative strategy that improves tobacco’s phytoextraction capacity and ensures edible leaves’ food safety.

Combing plants and their associated microorganisms to eliminate contaminants provides a cost-effective, in-situ, and promising technology [16]. The root-associated microorganisms, such as mycorrhizal fungi and endophytic fungi, can remove, inactivate, or degrade harmful environmental contaminants [17,18,19]. Endophytic fungi are the essential components of root microflora in the metal-contaminated ecosystem [20]. They possess various degradation pathways, such as metal sequestration and chelation systems, by which they increase host heavy metal tolerance and assist the host survival in contaminated soils [21,22,23]. During symbiosis, endophytic fungi either directly induce resistance of the host plants to deal with heavy metal toxicity or indirectly improve tolerance by improving water and mineral nutrient uptake in plants, increasing shoot biomass, causing modification in the root morphology. Endophytic fungi-assisted phytoremediation is a cost-effective and environment-friendly strategy [24]. *Piriformospora indica*, a well-studied root-colonizing endophytic fungus, was isolated from the woody shrubs *Prosopsis juliflora* and *Zizyphus nummularia* growing in the Indian Thar desert [25]. It was classified in *Hymenomycetes*, *Basidiomycota* [25], and can colonize many plants. *P. indica* confers various benefits to host plants, such as growth promotion, seed yield increase, and enhanced resistance/tolerance to biotic and abiotic stresses [26,27,28,29]. *P. indica* could improve the tolerance of host plants to heavy metals [30,31]. It possesses an excellent capacity to immobilize heavy metals in plant roots, which can be very promising in phytoremediation [30]. 

Although it has been recognized that *P. indica* plays a vital role in plant tolerance to heavy metals, the underlying mechanism remains obscure. This work aims to assess whether *P. indica* has the potential to confer Cd tolerance to tobacco cultivars. In addition, the effect of *P. indica* on Cd tissue accumulation and subcellular repartition in tobacco was investigated. Furthermore, we applied label-free quantitative proteomics to explore the underlying mechanism of *P. indica*-induced Cd tolerance in tobacco. Our findings will elucidate the mutualistic mechanism between tobacco and *P. indica* under Cd stress. 

## 2. Materials and Methods

### 2.1. Co-Cultivation of P. indica and Tobacco

Co-culturing *P. indica* and tobacco assayed the effect of *P. indica* on the Cd tolerance of tobacco in tissue culture bottles. Tobacco seeds were surface-sterilized in 1% sodium hypochlorite solution for 10 min, rinsed in sterile water. They were then planted in solid Murashige & Skoog medium and inoculated with *P. indica* strain plugs (one seed, one fungal plug per bottle). The plants inoculated with sterile plugs were the control. The plants were kept with a 16 h light/8 h dark photoperiod at 24/22 °C for 20 days. Then, all plants were transplanted and underwent hydroponic culture. The liquid medium contained 0 and 2 mg·L^−1^ Cd, respectively. Four treatment groups were designed: the control (Con), *P. indica*-treated (Pi), Cd-treated (HM), and *P. indica*- and Cd-treated (PH) groups. Each group contained 30 plants. After treatment for 30 days, the Cd content and growth parameters of the tobacco plants were measured. In addition, the chlorophyll content was measured by a SPAD-502 chlorophyll meter (Konica-Minolta, Osaka, Japan).

### 2.2. Endophytic Fungus, Cd Treatment, Microscopy

We evaluated the Cd tolerance of *P. indica* by inoculating a fungal plug onto a piece of sterilized glass paper placing on potato dextrose agar (PDA) containing Cd at a series of concentrations ranging from 0 to 2.5 mg·L^−1^. The fungus was incubated at 25 °C for 10 days in darkness. The MIC value was used to evaluate the tolerance of *P. indica* to Cd^2+^. 

The Cd-treated fungal sample was desorbed in 20 mM Na_2_EDTA for 15 min, washed with deionized water 3 times, and stained with Leadmium Green AM dye (Molecular Probes, Invitrogen, Carlsbad, CA, USA) in the dark. Then, the sample was stained by lipophilic endocytic dye FM4-64 for 30 min. Finally, distilled water was used to wash the samples 3 times to remove the excess fluorescent dye. The Cd-specific fluorescent probe was detected at 515 nm using an excitation wavelength of 488 nm. The fluorescence of FM4-64 was detected at 640–700 nm using an excitation wavelength of 514 nm under a LSM780 laser scanning confocal microscope.

### 2.3. Determination of Metal Contents and Electron Microscopy

The metal content was analyzed by ICP-MS (iCAP RQ, Thermofisher Scientific, Foster, CA, USA). For transmission electron microscopy (TEM), the samples were examined in the Hitachi H-7650 instrument at 60–80 kV. The samples were observed for scanning electron microscopy (SEM) under a ZEISS Gemini SEM 300 (Carl Zeiss, Oberkochen, Baden-Württemberg, Germany). 

The root samples of HM and PH groups were collected, embedded in resin, then sliced into 5 μm slices by a slicer (LKB Bromma ultratome pyramitome 11800, Stockholm, Sweden). Subsequently, the ultrathin section was map-scanned by a micro-confocal Raman spectrometer (inVia-Reflex 532/XYZ, Renishaw, London, UK).

### 2.4. Distribution of Cadmium in Subcellular Fractions of Leaves and Roots

Subcellular tissue fractions were extracted according to the method described by Wu et al. [32]. Firstly, 0.5 g of the tissues were homogenized in cold extracting buffer (50 mM Tris-HCl, 1.0 mM DTE (C_4_H_10_O_2_S_2_), 250 mM sucrose, 5.0 mM ascorbic acid, and 1.0% *w*:*v* Polyclar AT PVPP (pH = 7.5) with a chilled mortar and pestle. The homogenate was then filtered by a nylon cloth (240 μm). The cell wall residue on the nylon cloth was washed three times with the buffer. The filtrate was then centrifuged at 2500× *g* for 20 min (for root filtrate), or l500× *g* for 10 min (leaf filtrate). In addition, the pellet obtained was the chloroplast/trophoplast fraction for leaf and root, respectively. Then, the supernatant was centrifuged at 5000× *g* for 35 min, and the pellet was regarded as the membrane and organelle fraction, while the supernatant represented the soluble fraction. All steps were performed at 4 °C. Cd contents of all subcellular fractions were determined via ICP-MS described above.

### 2.5. Determination of Antioxidant Substances

The root samples of tobacco plants that underwent hydroponic culture for 30 days were collected for analysis. The activities of the antioxidant enzyme POD and the level of GSH were assessed. According to the manufacturer’s protocols, all assays were conducted using commercial chemical assay kits (Nanjing Jiancheng Bioengineering Institute, Nanjing, China), and each sample was examined in triplicate.

### 2.6. Protein Preparation, Mass Spectrometry, Identification, and Quantification

Total proteins of 300 mg of fresh roots were extracted by a modified trichloroacetic acid (TCA)-acetone-phenol method [33]. In brief, 100 μg of the protein were digested using the filter-aided sample preparation (FASP) method. The peptides were then desalted by ZIPTIP C18 spin tips (Millipore, Merck KGaA, Darmstadt, Germany). A liquid chromatography/mass spectrometry (LC/MS) system analyzed the tryptic peptides [34,35,36].

MaxQuant_1.5.2.8 analyzed raw MS files. The databases of *P. indica* (proteome ID: UP000007148) and tobacco proteins were interrogated. A pairwise comparison of HM and PH samples in triplicate was performed to explore the similarities and differences. We used label-free quantification to identify differentially expressed proteins (DEPs) with a 95% confidence level (*p*-value ≤ 0.05), and fold change > 2. ANOVA calculated the significance. Mapping and annotation were performed with Blast2GO (http://www.ebi.ac.uk/interpro/InterProScanv5.22-61.0 (accessed on 23 January 2017). DEPs mapping and pathway analysis were performed through the KEGG pathway database (http://www.genome.jp/kegg/Release85.0) (accessed on 1 January 2018). The StringDB protein interaction database (http://string-db.org/v.10) (accessed on 16 April 2016 to 14 May 2017) was used for the identified protein-protein interaction (PPI) analysis.

### 2.7. Statistical Analysis

Each experiment was performed in triplicate. The data were statistically analyzed using SPSS version 16.0 software (SPSS Inc., Chicago, IL, USA) and are presented as the mean ± standard deviation (SD). Graphs were created using GraphPad Prism 8.

## 3. Results

### 3.1. P. indica Improves the Cd Tolerance of Tobacco

**Figure 1:**
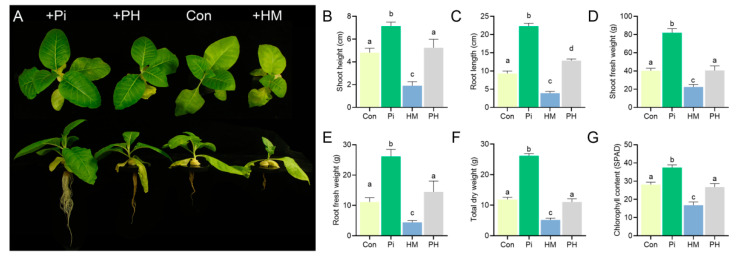
Cd tolerance of tobacco conferred by *P. indica*. (**A**) Effect of *P. indica* on the growth of tobacco seedlings in the presence of Cd. Control: non-treated, +F: *P. indica*-inoculated, +Cd: Cd-treated (5 μM), +CdF: both *P. indica*- and Cd-treated (5 μM). The same abbreviations are used below. (**B**–**G**) Effects of *P. indica* on the growth parameters of tobacco seedlings under Cd stress. The bars represent the means ± SDs, *n* = 9. Significant differences (one-way ANOVA): lowercase letters (a–c) mean *p* < 0.05.

The presence of *P. indica* significantly promoted plant growth. Compared with the control group, the growth parameters of *P. indica*-colonized seedlings, such as the shoot height, shoot fresh weight, root length, fresh root weight, and total dry weight, were enhanced by 48.54%, 103.63%, 139.16%, 134.77%, and 120.39%, respectively (*p* < 0.01) (Figure 1A–F). Cd treatment resulted in tobacco seedlings growing slowly and turning yellow (Figure 1A). However, the root colonization of *P. indica* alleviated these toxicity symptoms. The *P. indica*-colonized tobacco seedlings grew normally without any apparent dwarfing or chlorosis (Figure 1A), showing a higher level in the shoot height (173.82%), shoot fresh weight (227.38%), root length (228.13%), root biomass (228.11%), and chlorophyll content (59.4%) than Cd-treated plants (*p* < 0.01) (Figure 1B–G). These results suggested that *P. indica* conferred an improved Cd tolerance to tobacco. 

### 3.2. P. indica Alleviates the Root Damage Caused by Cd

**Figure 2:**
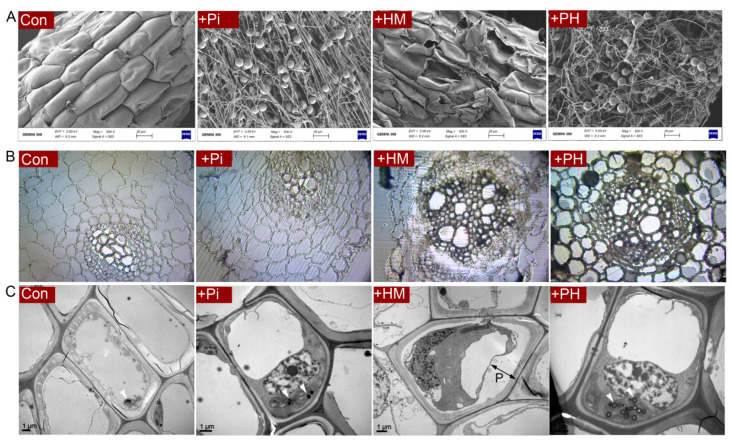
(**A**) Scanning electron microscopy of the root surface. (**B**) Observation of semithin section of root cross-section. (**C**) Ultrastructure of root cells under transmission electron microscopy. *p* means plasmolysis. Arrowheads mean plastoglobulis.

SEM observations showed that the root surface of the control seedlings was intact, while the root surface of the Cd-treated group was severely damaged. In the natural condition, *P. indica* formed many running hyphae and chlamydospores wrapping around the root surface. The hyphae grew longitudinally and were arranged neatly, whereas, under Cd stress, fungal hyphae became twisted and deformed (Figure 2A). 

The semithin section showed that Cd stress resulted in severe shrinkage and deformation of root epidermal and cortical cells. However, *P. indica* colonization eliminated these toxic symptoms caused by Cd, arranging the root cells to line up tightly (Figure 2B).

Ultrastructural observations further revealed that Cd exposure resulted in severe plasmolysis in root cells (Figure 2C). However, the presence of *P. indica* eliminated this plasmolysis, with the cells showing smooth and continuous cell membranes and a granular cytoplasm (Figure 2C). 

### 3.3. Effect of P. indica on Cd Accumulation, Subcellular Distribution, and Antioxidant Level

**Figure 3:**
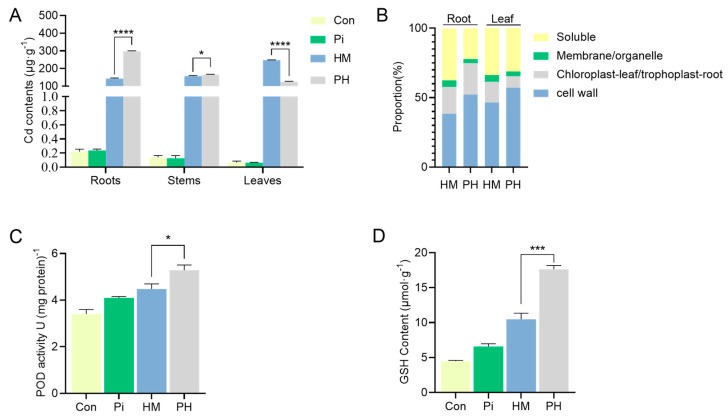
(**A**) Effect of *P. indica* on the spatial accumulation of Cd in different tissues of tobacco seedlings. The bars represent the means ± SDs, *n* = 3. Different letters on the bars mean significant differences at *p* < 0.001 (*t*-test). (**B**) The subcellular proportion of Cd in tobacco roots and leaves with *P. indica* inoculation or not. (**C**,**D**) The effect of *P. indica* on the antioxidant levels in tobacco under Cd stress. The bars represent the means ± SDs, *n* = 3. Significant differences (*t*-test): * means *p* < 0.05, *** means *p* < 0.001, **** means *p* < 0.0001.

The improved Cd tolerance of tobacco conferred by *P. indica* prompted us to investigate whether the accumulation and distribution of Cd in tobacco were altered. It was found that the presence of *P. indica* significantly increased the Cd concentration in roots (by 108.73%) and decreased the Cd concentration in leaves (by 47.42%) compared to the non-inoculated tobacco under Cd treatment (*p* < 0.0001) (Figure 3A). The majority of the Cd was accumulated in the leaves of HM group plants. However, the colonization of *P. indica* altered this accumulation pattern, accumulating most Cd in the roots of PH treatment plants while reducing Cd accumulation in leaves (Figure 3A). The subcellular repartition of Cd in tobacco roots and leaves was investigated for HM and PH treatments (Table 1, Figure 3B). Across all treatments, the accumulation of Cd in the cell walls was the highest in both leaves and roots, followed by the soluble fractions, and the accumulation of Cd in the membrane/organelle fraction was the lowest (Figure 3B). Notably, the Cd concentrations in all subcellular components of the *P. indica*-inoculated plant roots were significantly higher than those in the non-inoculated plants. On the contrary, the Cd concentration in each component of the *P. indica*-inoculated plant leaves significantly decreased compared to the non-inoculated plant (Table 1).

Interestingly, *P. indica* colonization increased Cd accumulation in the cell wall, which resulted in a significant decrease in Cd accumulation in membrane/organelle and soluble fractions in both leaves and roots (Figure 3B). For instance, under Cd stress, the proportion of Cd accumulation in the cell wall of roots and leaves of non-inoculated tobacco was 38.43% and 46.55%, respectively. In comparison, the proportion of Cd accumulation in the cell wall of roots and leaves in *P. indica*-inoculated plants was 52.23% and 57.11%, respectively (Figure 3B). The proportion of soluble Cd in *P. indica*-colonized roots decreased from 37.58% to 22.28% and from 33.83% to 31.25% in leaves. Similarly, the proportion of Cd accumulation in membrane/organelle components decreased from 4.74% to 2.89% in roots and from 4.60% to 3.29% in the leaves, respectively (Figure 3B).

Antioxidants have been reported to have the ability to alleviate heavy metal toxicity. Thus, we determined whether the antioxidant status was altered in *P. indica*-colonized tobacco to cope with Cd^2+^ toxicity. Under Cd stress, the POD activity in *P. indica*-colonized tobacco reached 5.29 U (mg protein)^−1^, increasing by 18.08% compared with non-colonized tobacco (Figure 3C). Concomitantly, the GSH content of the PH group was 17.62 μmol·g^−1^, 68.13% higher than that of the HM group. (Figure 3D). Overall, the findings indicated that *P. indica* elevated antioxidant levels as a detoxification strategy to neutralize Cd toxicity.

### 3.4. Effect of P. indica on GSH Distribution in Root Cells

**Figure 4:**
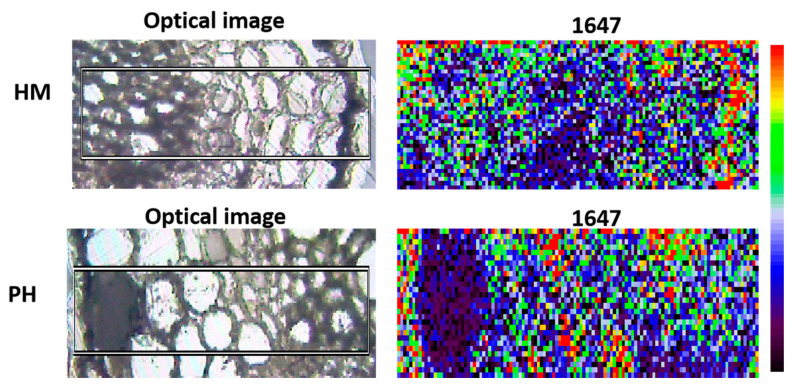
Raman microscopic images of GSH distribution in HM and PH root samples. Wave number = 1647. Red indicates high concentration. Blue indicates low concentration.

GSH plays a central role in scavenging reactive oxygen species (ROS) to maintain redox homeostasis [37]. Sánchez-Illana had determined the GSH peaks by Raman spectra [38]. Among the wave numbers, a wavelength of 1647 was selected to visualize the GSH distribution. It showed that the colonization of *P. indica* changed the spatial distribution of the GSH in tobacco roots under Cd stress. GSH was concentrated in the cortical cells in *P. indica*-inoculation roots while in the epidermis of the non-inoculation roots (Figure 4).

### 3.5. P. indica Can Absorb and Accumulate Cd

**Figure 5:**
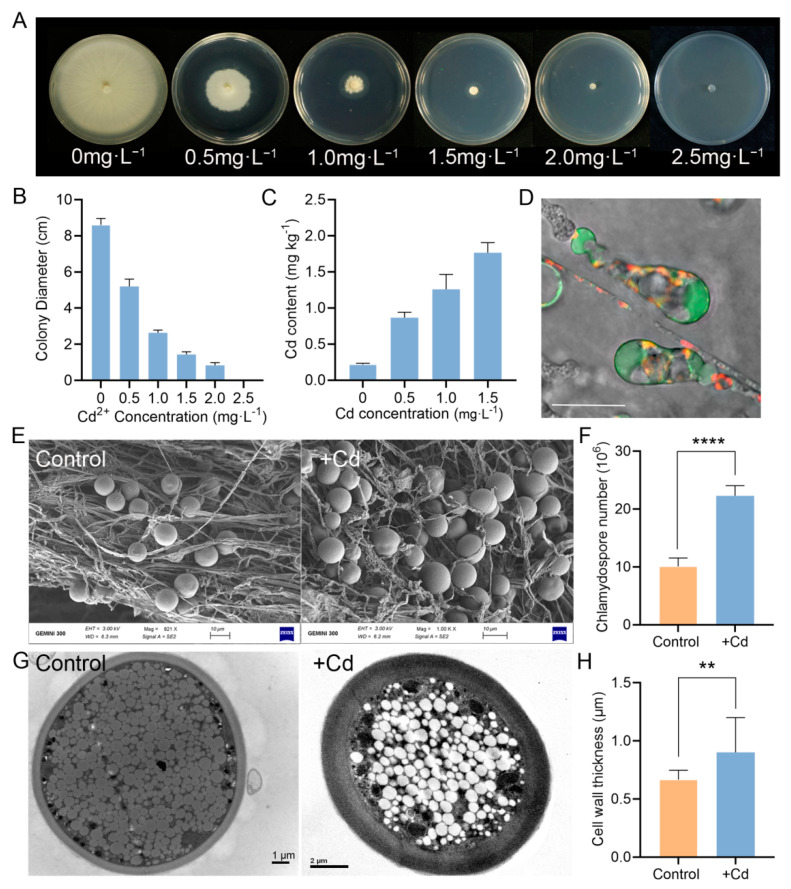
Cd tolerance assay of *P. indica*. (**A**) Colony morphology of *P. indica* exposed to different concentrations of Cd. (**B**) Fungal growth of *P. indica* cultured on different concentrations of Cd for 10 days. (**C**) The Cd accumulation in *P. indica*. (**D**) Cellular Cd^2+^ distribution in *P. indica* grown in PDA supplemented with 0.5 mg·L^−1^ Cd for 10 days. Scale bars = 10 μm. (**E**) Comparison of the fungal morphology of *P. indica* treated with 0 and 0.5 mg·L^−1^ of Cd by SEM microscopy. (**F**) Effect of Cd on chlamydospore production. *P. indica* was treated with 0 and 0.5 mg·L^−1^ of Cd, respectively. (**G**) Scanning electron microscopy of chlamydospores. (**H**) Effect of Cd on chlamydospore cell wall thickness. *P. indica* was treated with 0 and 0.5 mg·L^−1^ of Cd, respectively. The bars represent the means ± SDs. Significant differences (*t*-test): ** means *p* < 0.01. **** means *p* < 0.0001.

Enhanced Cd tolerance conferred by *P. indica* has prompted us to investigate the underlying mechanism. Does *P. indica* protect tobacco from Cd toxicity by absorbing Cd into its body or neutralizing Cd or by inducing resistance in tobacco? Therefore, we first evaluated the cadmium tolerance of *P. indica* through culturing *P. indica* under a Cd gradient. When the Cd^2+^ concentration reached 2.5 mg·L^−1^, fungal colony growth was inhibited entirely (Figure 5A). Thus, the minimum inhibitory concentration (MIC) value for *P. indica* was only 2.5 mg·L^−1^, indicating that *P. indica* possessed the ability to absorb Cd, but its tolerance is not high (Figure 5B). 

Along the Cd gradient, the Cd content in mycelia increased from 0.217 to 1.765 mg·kg^−1^ (Figure 5C). We further monitored the cellular distribution of Cd^2+^ by specific Cd^2+^ fluorescent dyes. It was found that the Cd^2+^ fluorescence signal was mainly concentrated in vacuoles of hyphae and chlamydospores (Figure 5D), suggesting that the vacuoles were the compartment to sequester Cd^2+^.

Under Cd stress, *P. indica* hyphae twisted (Figure 5E), and the number of chlamydospores significantly increased (*p* < 0.0001) (Figure 5E,F). The subcellular structures of *P. indica* under Cd stress were observed by TEM. The cell walls of chlamydospores were significantly thickened under the stress of Cd (*p* < 0.01) (Figure 5G,H). 

### 3.6. Quantitative Proteomics of Tobacco and P. indica Interaction under Cd Stress

**Figure 6:**
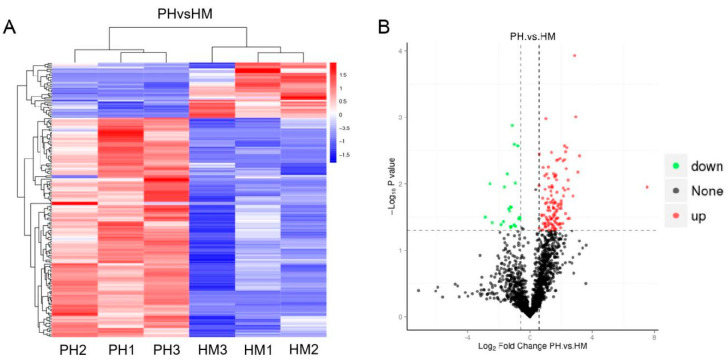
Differentially expressed proteins (DEPs) in Cd-treated tobacco plants inoculated with and without *P. indica*. (**A**) Heatmap of DEPs between HM and PH groups. Red, increase in expression abundance; blue, decrease in expression. Con1, Con2, and Con3 represent the control groups; Cd-1, Cd-2, and Cd-3 represent the Cd-treated groups. The horizontal axis represents the sample clusters. Similarity increases with decreasing cluster length. The clusters on the vertical axis indicate the expression modes of the clusters of proteins between PH and HM groups. The red and blue segments indicate relatively upregulated and downregulated protein expression, respectively. HM: Cd-treated plants; PH: both Cd-treated and *P. indica*-inoculated plants. (**B**) Volcano plot of the DEPs between the PH and HM groups. Red indicates significantly upregulated proteins. Green indicates significantly downregulated proteins. Black indicates proteins whose levels did not significantly differ between the two groups.

Since the tolerance of *P. indica* to cadmium is low, it is speculated that *P. indica* could induce resistance of tobacco to Cd stress. Then, we conducted high-throughput label-free quantitative proteomics to compare the HM and PH tobacco plants. A total of 183 proteins were identified as differentially expressed proteins (DEPs) with a confidence of >95% (Figure 6A, Table S1). Of these, 146 proteins were upregulated, and 37 proteins were downregulated (Figure 6B). Among these 183 proteins, 178 were plant proteins, and 5 were fungal proteins from *P. indica* (expressed inside the host plant roots). These five fungal proteins were actin-related protein (CCA73814.1), F-type H ^+^-transporting ATPase subunit (CCA68781.1), FoF1-type ATP synthase alpha subunit (CCA74650.1), histone H3 (CCA72783.1), and F-box-WD40 repeat protein (CCA74347.1), respectively (Table S1). It is interesting to note that the five fungal proteins were uniformly upregulated (Table S1). Among the upregulated proteins, peroxidase proteins accounted for the highest proportion, with ten proteins. The second was photosystem II proteins. In addition, one thioredoxin 1 protein and one glutamine synthetase protein were also significantly upregulated (Table S1). 

One hierarchical clustering showing differentially expressed proteins in HM and PH groups indicates that the root colonization by *P. indica* induced large changes in protein levels. Many significant DEPs were implicated in energy production and conversion, cellular processes, and signaling during Cd exposure (Table S1).

### 3.7. Functional Classification of Differentially Expressed Protein 

**Figure 7:**
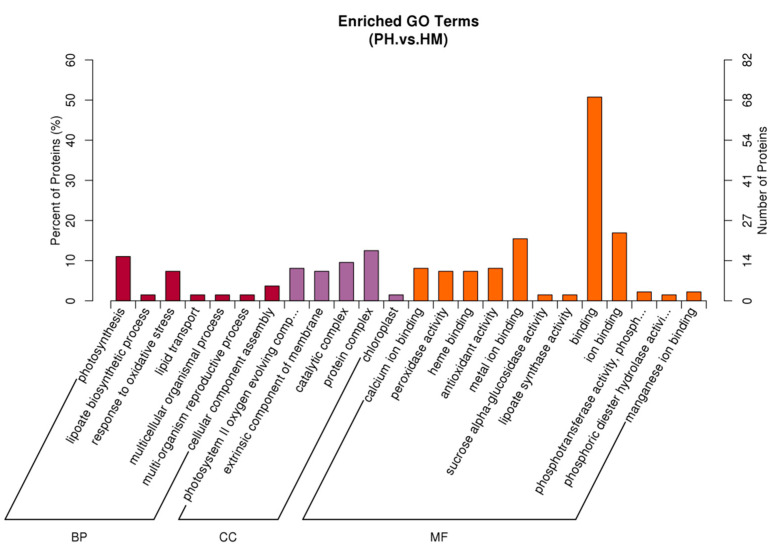
Gene Ontology analysis of the DEPs enriched in tobacco inoculated with/without *P. indica* during exposure to Cd (HM vs PH). GO is divided into three categories: (1) Biological Process (BP), which describes the biological processes of protein products; (2) Cellular Component (CC), which describes subcellular structure and location and macromolecular complexes; and (3) Molecular Function (MF), which describes the functions of proteins and their products.

The Gene Ontology (GO) classification was performed for the identified differentially expressed proteins of the tobacco plant (Figure 7). A total of 136 out of 183 DEPs were annotated. The DEPs in the HM and PH groups covered a wide range of molecular functions (MF), biological processes (BP), and cellular components (CC). It was found that the major proportions of 50.74% (69/136) of proteins in the MF category were involved in the binding, which are essential components of biosynthesis, degradation, and energy-related reactions. In addition, 16.91% (23/136) of proteins engaged in ion binding, and 15.44% (21/136) of proteins engaged in metal iron-binding. In the BP category, 11.03% (15/136) of proteins engaged in photosynthesis, and 7.35% (10/136) of proteins were involved in response to oxidative stress. In the CC category, most proteins were concentrated in the photosystem II oxygen evolving complex (15/136), catalytic complex (13/136), extrinsic component of membrane (10/136), protein complex (17/136), membrane (15/136), chloroplast (2/136). The rest of proteins are related to other biological regulation processes, such as antioxidant activity (11/136), peroxidase activity (10/136), response to stress (12/136), calcium ion binding (11/136), lipoate biosynthetic process (2/136), lipoate synthase activity (2/136), and iron-sulfur cluster binding (1/136), etc.

**Figure 8:**
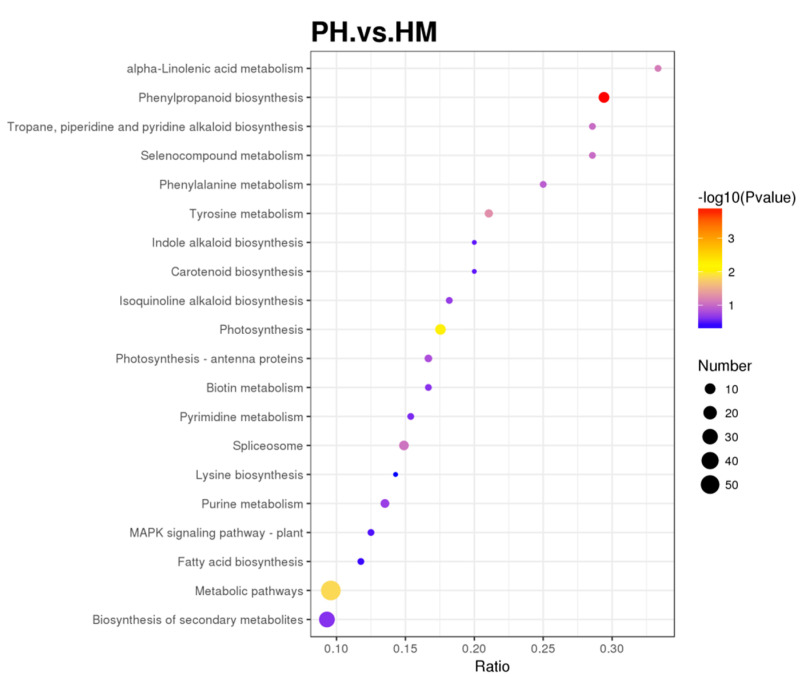
Kyoto Encyclopaedia of Genes and Genomes (KEGG) of the differentially expressed proteins (DEPs) between HM and PH groups. The horizontal axis represents the ratios of the number of DEPs in the corresponding pathways to the number of proteins detected. The DEPs enrichment level in each pathway increased with ratio magnitude. Dot color represents the *p*-value verified by a hypergeometric test. Test reliability and statistical significance increased with decreasing *p*-value. Dot size represents the number of DEPs in the corresponding pathway. The number of DEPs in the pathway increased with the total number of DEPs.

These biological pathways are also mapped in KEGG and were annotated in 20 KEGG pathways (Figure 8, Table S2). The KEGG pathway enrichment analysis determined the main metabolic and signal transduction pathways of the DEPs between the HM and PH groups. In addition, a bubble chart of the KEGG pathway was plotted. The KEGG pathway enrichment analysis indicated that the DEPs between the HM and PH groups were mainly related to phenylpropanoid biosynthesis (10 DEPs, *p* < 0.001), photosynthesis (10 DEPs, *p* < 0.01), and metabolic pathways (57 DEPs, *p* < 0.05). 

**Figure 9:**
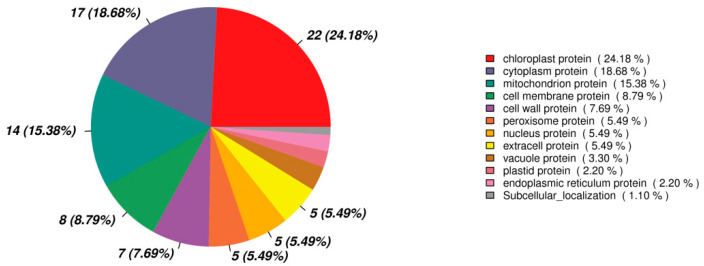
Subcellular localization of DEPs (HM vs PH).

Among the 183 DEPs between the HM and PH groups, 90 DEPs were annotated with a location. Most of the DEPs proteins were located in the chloroplast, accounting for 24.18%, followed by the cytoplasm protein (18.68%) and mitochondrion protein (15.38%) (Figure 9). Cell membrane and cell wall proteins accounted for 8.79% and 7.69%, respectively (Figure 9). It was suggested that the expression of the proteins on these organelles changed significantly in response to Cd stress.

### 3.8. Protein-Protein Interaction (PPI) Analysis

**Figure 10:**
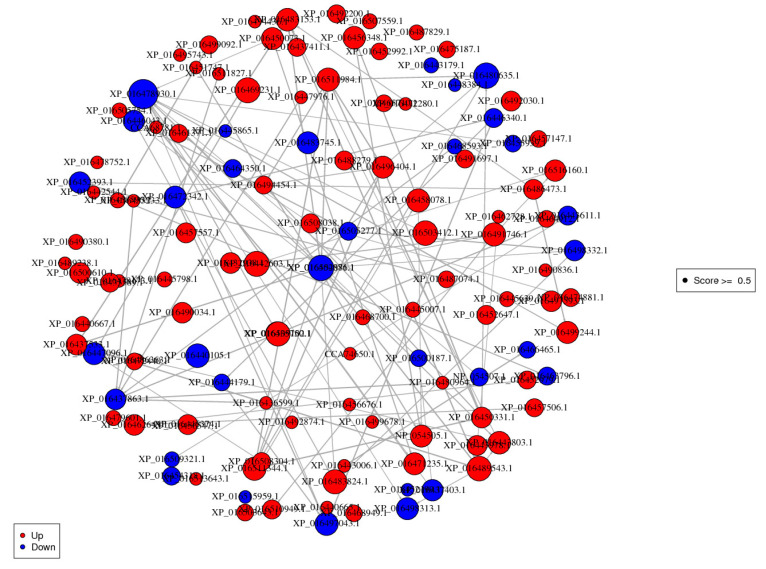
Protein-protein interaction networks of differentially expressed proteins. Red indicates significantly upregulated proteins. Blue indicates significantly downregulated proteins.

To explore the putative function and relationship of the Cd resistance responsive proteins induced by *P. indica*, we analyzed the functional overview of the DEPs using Cytoscape v2.8.3 software (confidence scores ≥ 0.5). Figure 10 and Table S3 depicted the densely connected network, in which 98 DEPs were mapped to the tobacco protein-interactome database, and one protein was mapped to the *P. indica* protein-interactome database. The proteins centrally concentrated in translation, ribosomal structure and biogenesis, secondary metabolites biosynthesis, transport and catabolism, and energy production and conversion in this network. The findings were consistent with our GO enrichment and KEGG pathway analysis.

## 4. Discussion

Heavy metals usually have toxic effects on plants, such as low biomass accumulation, chlorosis, inhibition of growth and photosynthesis, changes in water balance and nutrient assimilation, senescence, etc., eventually leading to plant death [39]. In the present study, toxicity symptoms caused by Cd in tobacco were notable, such as chlorosis, dwarfing, reduced biomass, and root tissue damage. However, the root colonization of *P. indica* significantly alleviated the harmful effects of Cd and promoted the growth of tobacco. Furthermore, *P. indica* colonization increased the Cd accumulation in tobacco roots, thereby alleviating Cd toxicity, and decreased Cd accumulation in shoots. Therefore, it suggests that *P. indica* confers a marked Cd tolerance to its host plant and plays a functional role as a beneficial plant-associated fungus. Recently, *P. indica* has been extensively applied for alleviating heavy metal toxicity in kinds of plants, such as wheat [40], sunflower [30], *Cassia angustifolia* [41], and rice [42,43]. Shahabivand et al. [30] reported that *P. indica* inoculation also inhibited the transportation of Cd from roots to aerial tissues in sunflowers. 

The detoxification capacity of plants partly depends on how they distribute toxins in their tissues [44]. The distribution pattern of metal ions in plant tissues is related to their toxicity and is vital for plant survival under toxic metal stress [45]. Cd can be accumulated in specific subcellular sites, such as vacuoles or cell walls, where Cd might be insolubly complexed to limit the translocation of Cd^2+^ strongly. Cell walls contain proteins and polysaccharides as ligands for binding metals and act as the first barrier against heavy metal entry into cells [46]. Therefore, plants bind heavy metals on the cell wall, which blocks the transmembrane transport of heavy metals to protoplasts to maintain the normal metabolism of plant cells [47]. Excessive accumulation of heavy metals in the plant membrane will damage cell activity and thus inhibit plant growth [48,49]. The current study showed that *P. indica* colonization modified a distinct subcellular alteration of Cd accumulation in both roots and leaves, significantly increasing the proportion of cell-wall-bound Cd. Simultaneously, a significant reduction in Cd accumulation in the other subcellular fractions, especially membrane/organelle and soluble-Cd, alleviated the adverse effects of excessive Cd accumulation in both leaves and roots of *P. indica*-inoculated tobacco. It is suggested that *P. indica* enhanced the capacity of binding metal ions in the cell walls of roots and shoots. Arbuscular mycorrhizal fungus *Glomus intraradices* protected alfalfa (*Medicago sativa* L.) from Cd toxicity by increasing the proportion of Cd in cell walls, and reducing the proportion in organelles/membranes [44]. Dark septate endophyte *Exophiala pisciphila* distributed Cd into the cell walls and changed Cd^2+^ into inactive forms; thus, the host plant grew normally even under Cd stress [45]. Taken together, *P. indica* improved the Cd tolerance of tobacco by regulating the repartition of Cd in subcellular components. *P. indica*-colonized tobacco plants employ cell walls as the primary reservoir for Cd. 

Fungi-assisted phytoremediation of heavy metals is by two ways: directly (through fungal absorption) or indirectly (through improving tolerance in the host plant) [19]. Fungi possess unique tolerance mechanisms to overcome heavy metal stress, such as chelation of extracellular heavy metals, binding of heavy metals on cell walls, complexation, and segregation of intracellular heavy metals [50]. In the present study, *P. indica* formed abundant chlamydospores with thickened cell walls to withstand the Cd toxicity. *P. indica* accumulated Cd^2+^ in chlamydospores and employed vacuole sequestration as a survival strategy under Cd stress. Accumulation of heavy metals by spores has been found in many AM fungi, which is a way for sequestering heavy metals and protecting plants from heavy metal stress [51,52]. Additionally, Mohd et al. [42] also reported that *P. indica* accumulated arsenic in vacuoles and cell walls of spores. Overall, our findings indicated that *P. indica* served as a biofilter sequestering Cd^2+^ in its vacuoles, prevented Cd^2+^ from being absorbed by the tobacco, immobilized Cd in the roots, and reduced the Cd accumulation in leaves.

**Figure 11:**
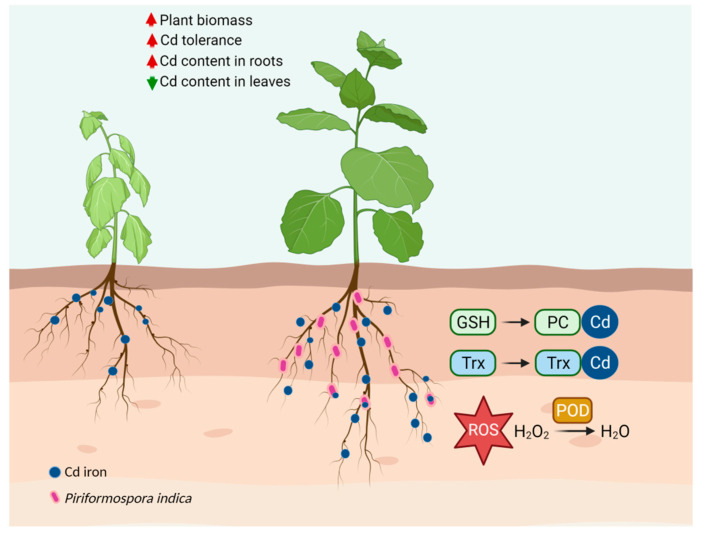
Schematic illustration of the mechanism for Cd detoxification in tobacco induced by *P. indica*. *P. indica*-inoculated tobacco possesses a sophisticated Cd detoxification network, including GSH, POD, and thioredoxin (Trx). Metal ions are shown as dark blue granular. *P. indica* is shown as pink granular.

Besides its accumulation capacity of Cd, *P. indica* also induced tobacco resistance to alleviate Cd toxicity. A symbiont of *P. indica*-tobacco possesses a sophisticated and interrelated detoxification network for Cd detoxification (Figure 11). According to the proteomics, the DEPs were mainly enriched in the oxidation-reduction process, carbohydrate and lipid metabolism, energy synthesis, and protein regulation during Cd exposure. These metabolic pathways were interconnected and endowed tobacco with tolerance to Cd through several aspects: (1) *P. indica* enhanced the resistance of tobacco to Cd by increasing the expression of POD. All plants produce various antioxidants and enzymatic scavengers to reduce the oxidative damage caused by Cd [53,54]. POD is a defense enzyme that works with superoxide dismutase and catalase to remove excess ROS caused by heavy metals. *P. indica* employed peroxidase to detoxify the Cd toxicity in tobacco. (2) *P. indica* enhanced the photosynthetic efficiency of tobacco and ensured energy supply even under Cd stress. It has been reported that *P. indica* improved photosynthetic pigments under arsenic [42], Cd [30], and copper [41]. Jogawat et al. [55] found that the colonization of *P. indica* increased the chlorophyll contents by upregulating the expression of the chlorophyll synthesis-related genes in rice seedlings under arsenic toxicity. Ghorbani et al. [56] reported that *P. indica* increased photosynthetic pigments to improve photosynthetic efficiency. Shahabivand et al. [30] also documented that *P. indica* promoted photosynthesis by improving electron transfer and the utilization of light energy under Cd toxicity. Cd stress also inhibited photosynthetic activities [57,58]. In the current work, tobacco plants turned to chlorosis under Cd stress. However, the *P. indica*-colonization in tobacco roots eliminated this chlorosis. Significant upregulation of the proteins involved in photosystem II (including photosystem II oxygen-evolving enhancer protein, photosystem II 13kDa protein, and photosystem II Psb27 protein) indicated that *P. indica* enhanced the photosynthesis in tobacco. Photosynthesis in *P. indica*-colonized tobacco leaves remained working normally even under Cd stress. (3) The enhancement of GSH regulated by glutathione synthase (GS) participated in Cd chelation in *P. indica*-colonized tobacco roots. Metal chelation is an essential detoxification mechanism for plants to combat heavy metal toxicity [59]. Chelation can be carried out by thiol compounds, such as glutathione [37], phytochelatins (PCs) [60], and metallothioneins (MTs) [61]. In particular, GSH participates in scavenging the accumulation of free radicals and reducing oxidative stress caused by heavy metals. Glutathione synthase (GS) catalyzed the biosynthesis of GSH. In *Arabidopsis thaliana* and *Schizosaccharomyces pombe*, the expression of GS was significantly increased in response to Cd [62,63]. Estrella-Gómez et al. [64] also showed that *Salvinia minima* plants increased resistance to Pb exposure by increasing GSH concentration, GS activity, and the SmGS expression level. Thus, GS is positively correlated with the heavy metal resistance of plants. This work found that the GS protein exhibited a higher accumulation in *P. indica*-inoculated tobacco roots, and the GSH content was enhanced and distributed in cortical cells. Thus, *P. indica* could help tobacco resist Cd toxicity by regulating the content and distribution of GSH. In addition, GS is also an essential enzyme for the assimilation and absorption of inorganic nitrogen. GS has been reported to enhance the biomass of some crops [65], including maize [66], wheat [67,68], and rice [69]. Therefore, the colonization of *P. indica* also enhanced the assimilation of inorganic nitrogen of tobacco roots, by which the host growth was promoted. (4) *P. indica*-colonized tobacco also employed a thioredoxin system to detoxify Cd toxicity. There are two thiol systems to maintain the cellular redox state in organisms. One is a low-molecular-weight thiol system, typically GSH [70]; the other is the thioredoxin system [71]. Thioredoxins can directly reduce or sequester heavy metals, and the resulting thioredoxin-heavy metal complex can be resolved by thioredoxin reductase leading to heavy metal detoxification [72]. Therefore, the enhanced Cd tolerance of tobacco induced by *P. indica* was correlated to the upregulation of thioredoxin protein.

## 5. Conclusions

In summary, *P. indica* partnered with tobacco roots improved host Cd tolerance, increased Cd content in tobacco roots by 108.73%, and reduced Cd content in leaves by 47.42%. *P. indica* accumulated Cd^2+^ into its chlamydospores and vacuoles, thereby preventing Cd^2+^ from being absorbed by the tobacco. *P. indica* increased the proportion of Cd in cell walls while reducing the proportion in organelles/membranes and soluble Cd. *P. indica* significantly enhanced the POD activity and GSH content in tobacco. Moreover, GSH was distributed in the cortex of *P. indica*-inoculated roots while in the epidermis of the control roots under Cd stress. The proteomics data showed that the proteins involved in peroxidase, GSH, and the thioredoxin system were enhanced by *P. indica* to detoxify the Cd toxicity in tobacco. Our findings indicate great potential for the application of this symbiosis in sustainable agriculture and phytoremediation. 

## Figures and Tables

**Table 1:** Subcellular distribution of Cd in tobacco inoculated with *P. indica* (PH) or not (HM) under Cd stress.

Tissue	Treatment	Cd Concentrations in Each Cell Fraction (μg·g^−1^)			
		Cell Wall	Trophoplast/Chloroplast	Membrane/Organelle	Soluble
Root	HM	54.29 ± 5.00	27.20 ± 0.71	6.70 ± 0.35	53.10 ± 1.77
	PH	155.63 ± 4.58 ****	67.37 ± 0.56 ****	8.61 ± 0.51 **	66.38 ± 2.89 **
Leaf	HM	115.93 ± 0.81	37.40 ± 0.45	11.47 ± 0.11	84.24 ± 0.83
	PH	71.87 ± 0.50 ****	10.50 ± 0.46 ****	4.14 ± 0.10 ****	39.32 ± 1.82 ****

The data are means ± SEM from three replications. ** indicates significant differences at *p* < 0.01, **** indicates significant differences at *p* < 0.0001 (by *t*-test).

## Data Availability

The data presented in this study are available on request from the corresponding author.

## Supplementary Materials

The following are available online at https://www.mdpi.com/article/10.3390/jof7080675/s1, Table S1: Differentially expressed proteins in PH vs. HM; Table S2: KEGG pathways; Table S3: Protein-protein interaction network.
